# Acute Oxalate Nephropathy Caused by Excessive Vegetable Juicing and Concomitant Volume Depletion

**DOI:** 10.1155/2022/4349673

**Published:** 2022-01-31

**Authors:** Harshad Chaudhari, Jennine Michaud, Nityasree Srialluri, Smita Mahendrakar, Christine Granz, Michael Yudd

**Affiliations:** ^1^Rutgers University Department of Nephrology and Hypertension, Newark, NJ, USA; ^2^Department of Veterans Affairs, Renal Section, East Orange, NJ, USA

## Abstract

Acute oxalate nephropathy (AON) induced by high dietary intake of oxalate-rich food is a rare cause of acute kidney injury and end-stage renal disease (ESRD). We describe a 68-year-old man with adequate baseline renal function who developed severe AON and ESRD. Six months earlier, he started a daily oxalate-rich fruit and vegetable juice diet high in spinach, with a calculated daily oxalate dietary intake of 1500 mg, about 10 times a typical diet. Renal biopsy showed extensive tubular oxalate deposits and acute tubular damage; the renal tissue was relatively free of chronic changes such as glomerulosclerosis, tubular atrophy, and interstitial fibrosis. A year later, he remains dialysis dependent.

## 1. Introduction

Oxalate is an end-product of glyoxalate metabolism in the liver. Diets rich in leafy vegetables and nuts also contribute oxalate and precursors of oxalate. Oxalate is cleared solely by the kidneys. We describe a patient with relatively good baseline renal function and free of microalbuminuria who developed severe acute oxalate nephropathy and end-stage renal disease because of excessive dietary intake of oxalate and volume depletion.

## 2. Case History

A 68-year-old Caucasian man with a medical history of insulin-dependent diabetes for 18 years and poorly controlled hypertension presented with weakness and was found to have acute kidney injury.

Six months before admission, he started a vegetable juicing regimen, thinking this diet was best for his overall health and may help with diabetic control. Hemoglobin A1c was 8.1 two months before admission and averaged 8.3 during the three years before admission. He blended one-third of a pound of raw baby spinach leaves with Swiss chard and added broccoli, cauliflower, green beans, asparagus, kale, beets, cilantro, parsnips, mushrooms, onions, and blueberries or raspberries. He added 4 ounces of water to the mix and drank one quart daily. For breakfast, he consumed oatmeal and vegetables. Lunch and dinner included pureed yams, spinach, Swiss chard, tofu, and garbanzo beans. He snacked on pumpkin seeds and pine nuts. His daily intake of oxalate was approximately 1500 mg/day, about 10 times that of a typical diet. This dietary oxalate amount was based on estimates of intake by the nutritionist and calculations utilizing the Harvard database [1]. Some of these foods had a very high oxalate-producing content, particularly spinach. Others did not.  Table 1 contains a list of the highest oxalate content foods consumed by the subject, along with the estimated oxalate content of his daily blended vegetable beverage.

He had had long-standing achalasia, which was treated with a myotomy and a Nissen fundoplication 16 years earlier. Associated with the achalasia, the patient had intermittent nausea, regurgitation, and vomiting for many years. However, 6 weeks before admission, these symptoms worsened, and he regurgitated small volumes about 6–8 times a day. He described having a poor appetite and an unintentional weight loss of 10 pounds. There was no history of chronic diarrhea, steatorrhea, or malabsorption.

An initial physical exam revealed a blood pressure of 180/72 mm Hg and heart rate of 55 beats per minute. His skin appeared to be dry with decreased turgor. The lungs were clear, the heart rhythm was regular with normal S1 and S2, and the abdomen was nontender with no peripheral edema. Antihypertension medications were amlodipine, lisinopril, doxazosin, and clonidine patch.

The initial labs showed a blood urea nitrogen (BUN) of 92 mg/dL and serum creatinine level of 9.6 mg/dL. The urinalysis performed by the nephrologist showed 1+ protein, a few clumps of white cells, and a moderate number of muddy brown casts. There were no crystals or bacteria. Two months and eight months before admission, serum creatinine was 1.4 mg/dl and the estimated glomerular filtration rate was 50 ml/min. Two months before admission, urine microalbumin was 8 mg/L, normal. Multiple microalbumin values before this were also normal over the previous 2 years.

He was aggressively hydrated, but the renal function did not improve. Hemodialysis was started, and a renal biopsy was performed. A 24 h urine collected one month after the patient started dialysis while he was ingesting a dialysate diet (2 g sodium, 2.5 g potassium, and 100 g protein diet) revealed a low urine oxalate level of 10 mg in 1400 ml of urine. He was in severe renal failure during the collection, so interpretation of this low oxalate is difficult.

**Figure 1:**
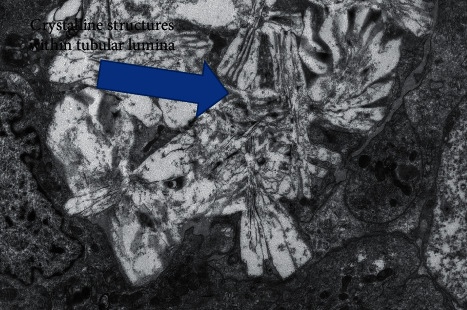
EM showing multiple oxalate crystals in the lumen of the kidney tubule.

**Figure 2:**
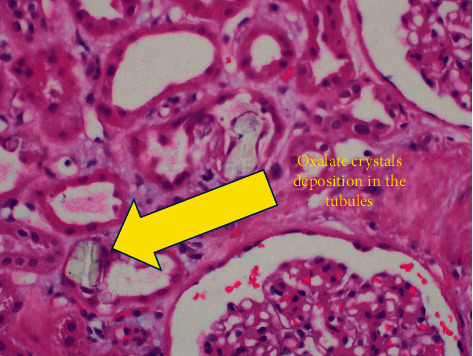
Oxalate crystals seen again in the kidney tubules.

The renal biopsy had 40 glomeruli, 3 of which were globally sclerosed. The glomeruli appeared normal in size and cellularity. The mesangial matrix was not expanded. Glomerular basement membranes were normal in thickness and contour. There was minimal chronicity noted in the tubulointerstitial compartments. There was acute tubular injury with simplification of the lining epithelium and dilatation of the lumen. Numerous and diffuse intratubular calcium oxalate deposits were noted (Figures 1 and 2), which demonstrated birefringence when viewed under polarizing light. There was severe arteriosclerosis and multifocal severe arteriolar hyalinosis.

The acute kidney injury was due to acute oxalate nephropathy. Upon further questioning, he denied ingestion of vitamin C or ethylene glycol, and he had no history of nephrolithiasis. Aside from the achalasia symptoms, he denied any other GI complaints or surgical procedures. There was no history of primary hyperoxaluria in his family. One year after this admission, he remained dialysis dependent.

## 3. Discussion

Oxalate nephropathy is classified as primary (genetic) or secondary (see Table 2). Primary hyperoxaluria (PH) is a rare collection of genetic diseases due to inborn errors of glyoxalate metabolism and characterized by overproduction of oxalate [2]. The genetic abnormalities of 3 hepatic enzymes can give heterogeneous clinical presentations for PH types 1, 2, and 3. PH type 1, usually the most severe, is the predominant type, making up 80% of the cases. The clinical manifestations of PH usually present early in life; the median age of onset for PH type 1 is 5.5 years, for type 2 is 3.2 years, and for type 3 is 2 years. However, cases of PH, particularly types 2 and 3, can present later in life, even for patients in their 70s [3]. The most common clinical picture is recurrent calcium oxalate renal stones. This can progress to nephrocalcinosis and CKD from oxalate deposits and, in the most severe cases, to oxalalosis, a diffuse systemic deposition of oxalate leading to a myriad of symptoms. A definitive diagnosis for PH requires genetic testing. Although we did not rule out PH, the clinical picture, an elderly man with no history of renal stones suddenly developing acute kidney injury from acute oxalate nephropathy, makes this diagnosis unlikely.

Secondary oxalate nephropathy is most commonly found in inflammatory small bowel disease, malabsorption syndromes, and intestinal bypass resections, known collectively as enteric hyperoxaluria [4, 5]. In these states, excessive oxalate is absorbed in the gut by several mechanisms. Increased gut luminal free fatty acids in these states increase colonic permeability to oxalates. Also, intestinal calcium binds to free fatty acids and biliary acids. This leaves more luminal oxalate unbound, which can then be absorbed. The increased oxalate that is absorbed will eventually be filtered and cleared by the kidneys, resulting in hyperoxaluria. Typical settings for this are Crohn's disease, celiac sprue, and pancreatic insufficiency. Ileal resection and Roux-en-Y gastric bypass surgery and treatment with orlistat, a bowel and pancreatic lipase inhibitor, have been complicated by oxalate nephropathy [6–8]. Our patient had no symptoms of malabsorption or intestinal disease.

Secondary oxalate nephropathy has been described in ethylene glycol toxicity and excessive ingestion of vitamin C. Both substances are metabolized to oxalate. Less commonly, oxalate nephropathy has been described in patients with excessive intake of plant-based foods high in oxalate, including the juicing of certain vegetables and fruits [4, 9, 10].

Oxalate nephropathy is a rare finding on renal biopsy. In a recent report of more than 7,000 renal biopsies over 25 years at one institution, only 65 (less than 1%) had calcium oxalate deposits causing renal failure, and of these cases, only 3 were attributed to diets rich in oxalate [10]. All three of those cases were in patients who also had some degree of CKD. A recent systematic review of secondary oxalate nephropathy found 33 cases of biopsy-proven oxalate nephropathy, which were attributed to diets rich in oxalate [4]. The primary sources of oxalate in those cases were ascorbic acid and starfruit. Other dietary sources included spinach, rhubarb, Chaga mushrooms, black tea, and nuts (i.e., peanuts, cashews, and almonds) [10–12].

Why only a small number of oxalate-rich consumers develop oxalate nephropathy is not known. The vegetarian diet is a popular diet, with millions of people worldwide safely consuming an oxalate-rich diet. Besides the high-oxalate dietary consumption, there must be other environmental or genetic factors working together to cause oxalate nephropathy. In our patient, intravascular volume depletion probably played a major role. We presume that, in the setting of volume depletion, the ensuing avid sodium and water reabsorption in the renal tubules led to higher concentrations of the excessive luminal oxalate and favored calcium oxalate precipitation in the distal tubules.

Dietary calcium influences the gut absorption of oxalate [13, 14]. Oxalate in the intestinal lumen binds with calcium; this bound oxalate is poorly absorbed, and it will be passed out with the stool. If dietary calcium is low, more luminal oxalate will be unbound, and therefore, oxalate absorption will be increased. In healthy adults eating a customary free-choice diet and then fed a controlled diet containing a 20-fold normal oxalate load (2200 mg) and an adequate calcium intake (1200 mg), daily urinary oxalate more than doubled, from 28 mg on the customary diet to 70 mg on the extremely-high-oxalate diet (normal daily urinary oxalate is up to 45 mg). When they were fed the same high-oxalate diet but now with a very-high-calcium diet (3800 mg), daily urinary oxalate was unchanged from the free-choice diet, 29 mg. [13]. Our patient's dietary calcium may have been very low; a low-calcium diet combined with a high-oxalate diet would lead to greater oxalate gut absorption and greater hyperoxaluria.

Increased gut absorption of oxalate may be present even in patients with apparently normal gastrointestinal systems. The increased oxalate absorption may result from hyperactivity of the main oxalate gut transporters, the anion exchangers belonging to the SLC-26 gene family [15].

Recent evidence has shed light on the role of gut bacteria in degrading intestinal oxalate, thereby decreasing intestinal absorption of oxalate and decreasing urinary oxalate. The human microbiota harbors bacteria known as the oxalobiome, which can encode an oxalate degradation pathway [16, 17]. The oxalobiome members include *Oxalobacter formigenes*, *Escherichia coli*, Bifidobacterium spp., and *Lactobacillus* spp. Alterations and loss of the oxalabiome members can lead to hyperoxaluria and an increase in calcium oxalate stones. Settings for this are antibiotic therapy, malabsorption, and intestinal bypass surgeries.

In summary, we present a case of acute oxalate nephropathy leading to acute kidney injury and end-stage renal disease due to excessive intake of oxalate-rich foods and intravascular volume depletion. It might be prudent for people with a tendency for volume depletion to avoid this diet.

## Figures and Tables

**Table 1:** Subject's estimated daily oxalate intake (food item/serving size/amount of oxalate).

Ingredients with the highest oxalate content included in the daily vegetable juicing regimen		
Food item	Estimated quantity (serving size)	Oxalate content
Raw baby spinach leaves	2 cups	1312 mg
Swiss chard	1 cup	347 mg
*Asparagus*	4–8 spears	6–12 mg
Beets	1 cup	152 mg
Parsnips	½ cup	15 mg
Raspberries	½ cup	24 mg
		Estimated total = 1862 mg
Additional potential high-oxalate dietary sources (not consumed consistently by the patient)		
Yams (pureed)	1-2 cups	28–56 mg
Cooked spinach	½-1 cup	755–1510 mg
Tofu	3.5 oz	13 mg
Pumpkin seeds	½ cup	19.5 mg

**Table 2:** Primary and secondary causes of hyperoxaluria.

Primary (inherited)
PH1: deficiency of alanine-glyoxylate aminotransferase
PH2: deficiency of glyoxylate reductase/hydroxypyruvate reductase
PH3: deficiency of 4-hydroxy-2-oxo-glutarate aldolase
Secondary (acquired)
Enteric hyperoxaluria: fat malabsorption states, pancreatic insufficiency, intestinal bypass surgeries, and orlistat
Ethylene glycol
Excessive vitamin C ingestion
Excessive ingestion of high-oxalate foods, in particular star fruit
Deficiency of oxalate-degrading bacteria from antibiotic use

## Data Availability

This is a case report, and no data were collected.
